# Teratogenic Effects of Topiramate in a Zebrafish Model

**DOI:** 10.3390/ijms18081721

**Published:** 2017-08-07

**Authors:** Yu-Heng Lai, Yu-Ju Ding, David Moses, Yau-Hung Chen

**Affiliations:** 1 Department of Chemistry, Chinese Culture University, No. 55 Huagang Road, Taipei 111, Taiwan; lyh21@ulive.pccu.edu.tw; 2 Department of Chemistry, Tamkang University, No. 151 Ying-chuan Road, Tamsui, New Taipei City 25137, Taiwan; dingfion@gmail.com (Y.-J.D.); dcmoses@yahoo.com (D.M.)

**Keywords:** topiramate, ceratobranchial defect, teratology, zebrafish

## Abstract

Topiramate is commonly used for treating epilepsy in both children and adults. Recent clinical data suggests that administration of topiramate to women during pregnancy increases the risk of oral clefts in their offspring. To better understand the potential effects of topiramate, we dosed adult female zebrafish with topiramate, and investigated the altered morphologies in adult females and their offspring. It showed that topiramate-treated female fish had reduced oocyte maturation, and the survival rates of their offspring were seriously decreased during embryogenesis. In addition, around 23% of offspring displayed cartilage malformation in the craniofacial area, such as loss of ceratobranchial cartilages as well as impaired ceratohyal, Meckel’s cartilage and ethmoid plate development. Moreover, mineralization of ceratohyal, Meckel’s cartilage, and vertebrae were downregulated during bone development. Taken together, we concluded that topiramate impaired oogenesis in the maternal reproductive system, and then caused offspring cartilage malformation or bone dysplasia.

## 1. Introduction

Topiramate, an antiepilepsy drug for both children and adults, was first approved by the United States Food and Drug Administration (FDA) in 1996. It is licensed for treatment of migraine prophylax and Lennox–Gastaut syndrome [1]. Statistically, epilepsy is the fourth most common neurological syndrome in the United States [2]. An estimated 2.2 million people in the United States have epilepsy, which means the prevalence rate is around 0.71%. Anti-epileptic drugs are still the most common method for treating epilepsy although surgery is used in some cases [3]. This suggested to us that the side effects of these drugs should not be ignored. Among epilepsy medications, topiramate, a derivative from naturally occurring d-fructose, shows great effect in blocking the spread of seizures. Previous studies have demonstrated several possible mechanisms for its antiepilepsy activity, including enhancement of gamma-Aminobutyric acid (GABA) -evoked whole-cell currents in cortical neuron through a benzodiazepine-insensitive pathway [4,5], inhibiting L-type calcium channels to control calcium influx and dendritic excitation [6], inhibiting (RS)-2-amino-3-(3-hydroxy-5-methylisoxazol-4-yl)propionate (AMPA) and kainate (KA) receptors in controlling calcium influx [7].

However, according to the FDA drug grading, topiramate is classified as Pregnancy Category D, which indicates positive evidence of human fetal risk, but also potential benefits from use of the drug in pregnant women [8]. Along with this classification, labels on topiramate now warn of the risks of developmental defects in newborns [9]. Analysis of two birth defect studies from 1997 to 2007 and from 1997 to 2009 suggests that the rate of oral clefts in infants was increased with the exposure of topiramate during the first trimester of pregnancy [10].

More and more attention is focused on fetal risk, attributable to the usage of medications during pregnancy, the safety of newer antiepileptics has been under scrutiny and research has evaluated their potential risks. Animal studies on developmental toxicity of topiramate have shown some fetal malformations [11]. During rat studies, embryonic toxicity effects, such as weight loss and structural change, were observed among the offspring when orally treated with a low dose (20 mg/kg) during pregnancy. However, the effects randomly occurred with different degrees due to discrepancy between individuals. Increasing frequency of limb malformation and physical developmental retardation were noted among the offspring under 400 mg/kg treatment. In addition, not only pup, but also maternal weight reduction was shown at 100 mg/kg and more clinical signs of maternal toxicity were seen at 400 mg/kg. Both fetal and maternal toxicity were persistent throughout the entire treatment [12]. Similar morphological effects on rabbit were also observed. Both fetal and maternal toxicity were observed under 35 mg/kg treatment and skeletal malformation was seen at 120 mg/kg in offspring [12]. 

Although the results of case-control studies and pre-clinical animal tests have suggested several fetal defects attributable to topiramate [13], no topiramate-induced oral cleft animal model has been generated and investigated. Using zebrafish as an animal model, numerous benefits have been discovered, such as a great yield of easily collected embryos, rapid development, and genetic similarity to humans; this makes zebrafish excellent for application in our studies [14]. A previous study has reported that zebrafish showed comparable responses with a mouse model during GABA depleted analysis on different antiepileptic drugs, including topiramate [15]. Moreover, larval zebrafish is a well-established platform for high-throughput screening of drugs and provides a connection to translate laboratory research to clinic application [16]. This suggested to us that zebrafish is a suitable model for elucidating the drug mechanism. In this study, we noticed that the reproductive ability of the mother fish was decreased after treatment with topiramate; we hypothesized that under-developed oocytes were ovulated during fertilization. Also, we observed severe developmental malformation of cartilage and bone and suggested a connection between the maternal passage of the topiramate effect in offspring. Through our findings, hopefully the correlation of topiramate-associated oral cleft will be elucidated and may translate into side-effect prevention in the near future.

## 2. Results

### 2.1. Topiramate Showed No Effect on Weight of Experimental Female Fish

One of the most common side effects of topiramate is loss of appetite and weight loss. To observe whether female fish show similar side effects to humans, a total of eight topiramate-treated females and two controls were weighted during the feeding period (Figure S1). At the beginning of the experiment, the average weight of the experimental group was 0.88 g (range 0.7–1.1 g); the control group was 0.85 g (range 0.8–0.9 g). The average weight of the experimental group increased to 0.96 g (range 0.9–1.1 g), which was 0.08 g more than before; while the control group reached 1.05 (range 1.0–1.1 g), which increased 0.2 g. Although a previous report showed a decrease in metabolic rate and body weight between groups that take topiramate, there was no significant statistical difference when comparing the weight of two zebrafish groups.

### 2.2. Topiramate Impairs the Maturation of Oogenesis

Generally, oocyte development in zebrafish has been divided into four stages based on oocyte size and the presence or location of specific subcellular components and described as follows [17,18]. The first stage of oocyte development is the primary growth stage, in which oocytes begin to grow and progress into the early stages of meiotic prophase. During the cortical alveolus stage (II), follicles become translucent and increase in size with the germinal vesicle at the center surrounded by cortical alveoli. In the vitellogenesis stage (III), vitellogenin is expressed and accumulated in the yolk, and specialized follicle cells (micropylar cells) are formed from this stage. Until stage IV, the mature oocyte stage, meiosis is arrested. As meiosis is reinitiated, the germinal vesicle migrates to the oocyte periphery, the nuclear envelope breaks down, and meiosis is again arrested at the secondary meiotic metaphase. When the egg is ovulated and transported in the ovarian lumen, the maturation process of oogenesis is completed.

**Figure 1:**
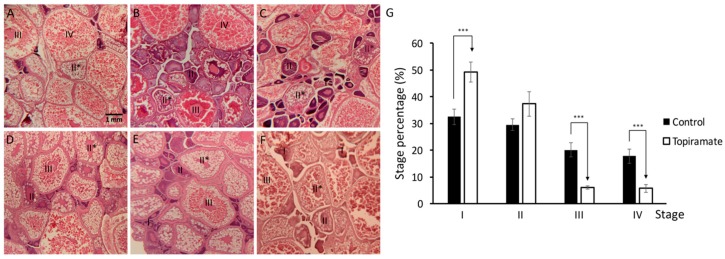
Under-maturation of oogenesis was shown in topiramate-treated female fish. Developmental status of oocyte in maternal fish was determined by Hematoxylin and eosin (H & E) staining. (**A**) Generally, the ovary was filled with mature oocytes in control fish in a high percentage; (**B**–**F**) however, a decreased percentage of mature oocytes was detected in different topiramate-treated female fish. Identification of the maturation stage was based on morphologies (I, primary growth stage; II, early cortical alveolus stage; II *, mid-cortical alveolus stage III, vitellogesis stage; IV, mature oocyte); (**G**) statistical analysis of oogenesis stages. Columns represent the number of cells at the four stages as percentages of the total counted (averages ± SD; *** *p* < 0.005; control *n* = 8; topiramate-treated *n* = 7).

Female fish were fed with topiramate for 7 days, and sacrificed to collect whole ovary after laying to eject mature oocytes. We hypothesized that oogenesis in mother fish is the topiramate-affecting stage during reproduction. According to the morphology of oogenesis stages, a large portion of ovarian tissue was filled with mature oocytes in the control group (Figure 1A). In contrast, a significant decrease in the percentage of mature oocytes was detected and the majority was at primary growth (I) and cortical alveolous (II) stages (Figure 1B–G). In addition, during the experiment, about 40% of females (*n* = 33) were able to lay eggs. This indicated that the uptake of topiramate in female fish affects oogenesis and suggested that it may lead to abnormal embryogenesis in offspring.

### 2.3. Topiramate Affects Epiboly Progression of Offspring Fish

**Figure 2:**
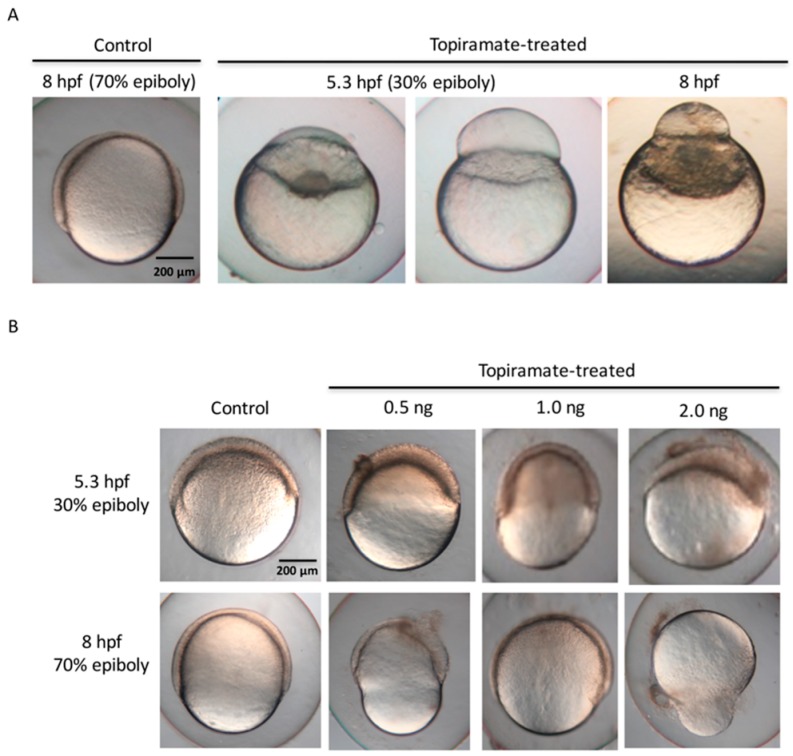
Embryonic epiboly deficiency in topiramate-treated offspring. (**A**) Embryonic development observation from control and topiramate-treated offspring. Morphology at 8 hpf in the control group showed normal epiboly progression. Embryos from topiramate-treated female fish were abnormally expressed during epiboly progression from 5.3 hpf; (**B**) embryonic development observation from control and topiramate-injected embryos at 5.3 and 8 hpf. Scale bar: 200 μm.

We next detected morphological alteration of F1 embryo of topiramate-treated fish. Embryos of 5.3 and 8 hpf (hour post fertilization) from topiramate-treated females were observed and the survival rate was recorded (Table 1). The survival rate of F1 embryos at 8 hpf was calculated and normalized over 5.3 hpf survival statuses. Embryos from topiramate-treated fish showed a lower survival rate than usual. Interestingly, an average of 16.3 ± 15.6% of early developmental malfunction, including aberrant epiboly migration at the 5.3 hpf stage and failure to differentiate, was detected in 8 hpf embryos (Figure 2A). According to morphology, the embryo was able to initiate epiboly progress with blastoderm formed. However, the enveloping layer of cells failed to migrate to the vegetal pole and accumulated at the animal pole, forming a bubble-like shape. In addition, blastoderm was unable to cover the yolk cell and halted before 50% coverage.

Generally, morphogenetic movement is mainly coordinated by cytoskeleton dynamics [19]. Microtubules and cortical actins create a network that is able to shepherd a series of cell rearrangements and movements. The first morphogenetic movement of gastrulation, named epiboly, is characterized by the migration of the blastoderm from the animal pole down to the vegetal pole [20]. In order to investigate whether mutation of the epiboly stage is due to the topiramate metabolite, we microinjected topiramate into the embryo during the 1- to 4-cell stage. Based on the assumption that topiramate dosage was absorbed in every egg laid from maternal fish, we injected 0.5, 1.0 and 2.0 ng drug between the boarder of the cell and the yolk of the embryo. Similar epiboly-halted phenomena of failure to complete epiboly progression were also observed at 5.3 and 8 hpf (Figure 2B). In addition, we found that the survival rate of 8 hpf embryos at 0.5 ng dosage of topiramate was 84%. When increasing the dosage to 1 ng, survival rates were decreased to 74%; while the survival rates were even lower (59%) under 2.0 ng dosage. The malformation ratio was also shown to have a dose-dependent effect, which was induced from 4.9% to 63.4% (Table 2). This may suggest that topiramate is transmittal, causing the epiboly deficiency.

### 2.4. Effect of Topiramate on Cartilage Development in Offspring

**Figure 3:**
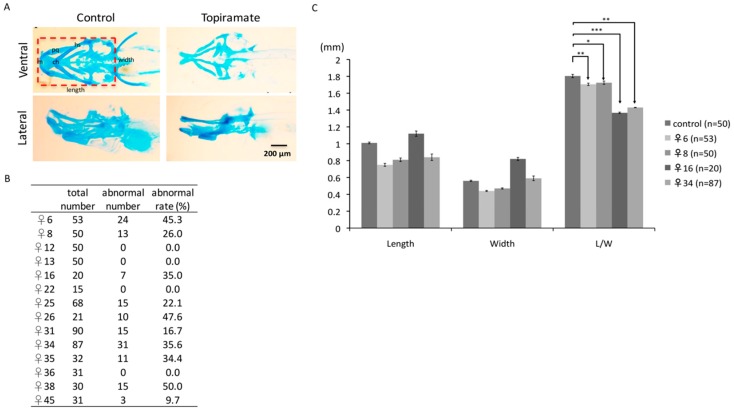
Cartilage development of offspring was impaired through maternal transmission of topiramate. (**A**) Ventral and lateral views of cartilage structure were detected by Alcian blue staining at 4 dpf larvae. Compared to the control group, there was a lack or shortage of cranial and pharyngeal development in topiramate-treated offspring; (**B**) embryonic number and abnormal rate were calculated from 14 female zebrafish; (**C**) length and width from individual maternal fish were measured and statistically analyzed. (averages ± SD; * *p* < 0.5; ** *p* < 0.01; *** *p* < 0.005; cb, ceratobranchial; ch, ceratohyal; ep, ethmoid plate; hs, hyosymplectic; M, Meckel’s cartilage; pq, palatoquadrate; Scale bar: 200 μm).

Cartilage and bone development have been well-studied in zebrafish. Cartilage is the predominant component in the cranium in early embryogenesis and is fully developed until 74 hpf [21]. Several studies have indicated that teratogenic drugs may affect cartilage and bone formations in early zebrafish developmental [22]. Therefore, we were interested in whether topiramate impairs the cartilage development. Alcian blue staining was performed to detect the cartilage of F1 fish at 4 dpf (days post fertilization). As the ventral view showed embryonic cranial and pharyngeal arrangement in the control group, we found transformation of Meckel’s cartilage (M), ceratohyal (ch), and ethmoid plate (ep) during embryogenesis in F1 fish (Figure 3A). Moreover, from the lateral section, ceratobranchial (cb) was found severely underdeveloped. While individual differences may cause divergent degrees of drug response, we did observe a similar under-developed status in the craniofacial cartilage region in diverse topiramte-treated offspring (Figure S2). However, almost complete cartilage development took place in some offspring. Only 14 fish were still fertile in 45 topiramate-treated females; among these 14 females, on average, 23% of F1 fish showed cartilage abnormality (Figure 3B). It is suggested that topiramate had a serious impact on cartilage development; teratogenic factors were indeed passed from mother to offspring.

### 2.5. Craniofacial Malformations on Topiramate-Treated Offspring

**Figure 4:**
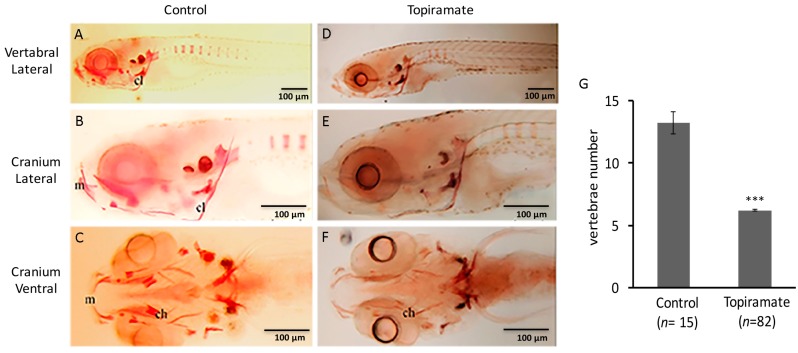
Bone development of offspring was impaired through maternal transmission of topiramate. (**A**–**F**) Ossification status of cranium and vertebral regions was detected by Alizarin red staining of zebrafish at 10 dpf; (**G**) Vertebrae numbers from offspring of control or topiramate-treated female zebrafish (♀25) (averages ± SD; *** *p* < 0.005; control *n* = 15; topiramate-treated *n* = 82). (ch, ceratohyal; cl, cleithrum; m, Meckel’s cartilage; Scale bar: 100 μm).

To confirm the craniofacial developmental defect in topiramate-exposed F1 offspring, we measured the ratio of Length/Width (*L*/*W*) in the cranium region as illustrated in Figure 3A. Compared with the control group, the F1 offspring from four topiramate-treated mother fish showed significantly decreased *L*/*W* within the craniofacial region, which suggested a larger ceratohyal angle (Figure 3C). Generally, ossification starts from the cranium region during embryogenesis at 5 dpf, while it extends to the spine at 7 dpf [23,24]. Therefore, skeleton formation status of 10 dpf F1 larvae was observed with Alizarin red staining to visualize whether cartilage abnormality affects endochondral ossification. From lateral and ventral views of fish without treatment, we can clearly examine the ossification status of cranium and vertebral regions (Figure 4). Compared to the control fish (Figure 4A–C), ossification reduction in the post-cranial axial skeleton (Figure 4D) and impaired ossification in the regions of ceratohyal and Meckel’s cartilage (Figure 4F) were observed. Moreover, the number of spinal segments was counted. On average, 13.2 spinal columns were developed in the control group; while it was 6.2 in topiramate-treated offspring (Figure 4G). To conclude, not only cartilage development, but also bone formation was impaired by topiramate treatment.

## 3. Discussion

Previously, teratologenic evaluation of topiramate in mice, rats, and rabbits has been reported and classified as a teratogen [11,25,26]. The reduction of weight and skeletal development were observed side effects of topiramate with maternal toxicity [11,25]. In addition, more case studies indicated a significant correlation between teratogenic effect and oral cleft in humans [10,13,27]. Therefore, understanding the relevance of topiramate treatment with craniofacial skeletal defects and mining the biological mechanism will be our goal now and in the future. To address the connection, we first focused on the reproductive defects of mother fish; then the maternal influence on skeletal development in offspring. In our study, we treated zebrafish with 0.5 mg/g/day of topiramate-blended dry food once per day for 7 days according to topiramate dosage for adult humans (500 mg/kg/day). On the other hand, while we validated the topiramate effect by direct injection into embryos, we calculated the dosage based on the assumption of absorption per clutch of eggs from topiramate-treated mother fish. Precise calculation was ensured so as not to overdose the fish and cause artificial toxicity and lead to experimental bias. Therefore, based on our assumption, the teratogenic effects are due to topiramate treatment. 

Hormone disturbance is known to be correlated with recurrence of epilepsy [28]. It is possible that infertility occurs in women who have epilepsy and this may be due to the side effects of antiepileptic medication. The fertility rate in women with epilepsy has been reported to be lower compared with their nonepileptic female siblings [29,30]. In fact, only 40% of maternal fish were fertile in our study. Generally, healthy female fish in our laboratory that reach maturation show relatively active fertility after light cycle stimulation until they become aged. Therefore, the decrease in fertility in topiramate-treated female zebrafish drew our attention and was an unusual phenomenon, which matches the topiramate-induced side effect in humans. Furthermore, the outcomes of endocrine dysregulation were abnormal menstrual cycle length and anovulatory [31]. It has also been published that rats treated with topiramate showed severe degeneration of placental structure and function and caused teratogenic defects [32]. This indicated that topiramate induced different degrees of congenital impairment not only producing under-maturated oocytes, but also through continuous maternal transmission from placenta to offspring. It also agreed with our result that the reproduction rate was reduced without detecting any significant weight loss in topiramate-treated zebrafish.

Craniofacial abnormality is one of the most common congenital birth defects, which affects the development of the head, face, and neck. According to the statistical data from the North American Antiepileptic Drug (NAAED) Pregnancy Registry, there may be a higher risk of oral cleft (1.4%) when infants are exposed to topiramate than other antiepileptic medications (0.38–0.55%) during the first trimester of pregnancy [8,10]. In our study, the ratio of zebrafish with cartilage malformation was about 23%, which is much higher than shown in a clinical study on humans. In fact, individual diversity may cause diverse degrees of drug response in fish, for example, only some human females who were dosed with topiramate showed various side effects, including offspring with an oral-cleft. Therefore, we observed that some of the offspring from certain female fish may show severe abnormality in craniofacial cartilage and others may not (Figure S2). Only few preclinical studies on rats and rabbits showed embryogenesis defects associated with topiramate; however, there was no solid research identifying the result of craniofacial defects linked with topiramate treatment and further investigation is definitely needed. Therefore, our study generated a topiramate-associated zebrafish model, which may provide a platform to identify the underlying mechanism of the myriad of fetal defects.

## 4. Materials and Methods

### 4.1. Zebrafish Maintenance

AB strain zebrafish were maintained under a 14 h on/10 h off light cycle as described in the Zebrafish Book [33]. Embryos were collected and cultured in petri dishes containing E3 water (5.0 mM NaCl, 0.17 mM KCl, 0.33 mM CaCl_2_ and 0.33 mM MgSO_4_) at 28 °C. The 1-phenyl 2-thiourea (PTU, p7629, Sigma-Aldrich, St. Louis, MO, USA) was added during embryogenesis to inhibit pigments formation. All animal experiments in this study were performed in accordance with the guidelines issued by the animal ethics committee of Tamkang University (Number: 106001; Issue date: 1 August 2016).

### 4.2. Topiramate Treatment

**Figure 5:**
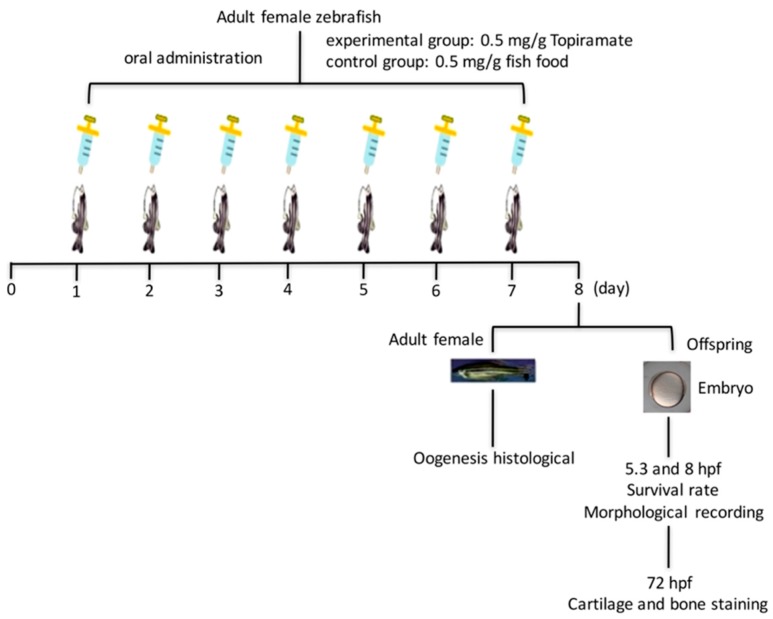
Experimental design scheme of topiramate treatment in zebrafish.

Adult female fish (around 4 months old) that had reached sexual maturity were chosen to undergo the topiramate-containing diet. Before beginning the dosage process, females were mated with males to completely ovulate eggs from their ovaries to begin a new cycle of oogenesis. In accordance with the topiramate dosage for adult humans (500 mg/kg/day), we fed fish 0.5 mg/g/day of topiramate-blended dry food once per day for a period of 7 days. At the end of the final day of drug feeding, we set up a breeding environment and collected embryos the following morning. The design of the topiramate treatment process is diagrammed in Figure 5. A total of 82 fish were treated during the whole study. Throughout the experiment, only 40% of females (*n* = 33) were able to lay eggs, as listed in Table 1 and Figure 3B for further observations.

### 4.3. Preparation and Staining of Paraffin Section

Animals were sacrificed in accordance with approved euthanasia techniques. Whole ovarian tissue was excised and fixed in 4% Paraformaldehyde (PFA) and rinsed in running water. Paraffin embedded blocks were sectioned at a thickness of 5 μm on a microtome. Slides were deparaffinized in a 55 °C water bath for 10 min, immersed in xylene two times for 10 min and then in different concentrations of ethanol (100%, 95%, and 85%) two times each for 5 min to rehydrate the tissue, and rinsed with distilled water before staining.

### 4.4. Hematoxylin and Eosin Staining

Deparaffinized and rehydrated slides were stained in hematoxylin for 3–5 min, washed in running water until sections turned blue, stained in 1% eosin Y for 10 min, dehydrated in 80%, 90%, and 100% ethanol for 20 s each, mounted and observed under a microscope. 

### 4.5. Tissue Staining

Alcian blue and alinzarin red stainings were performed as described [34]. Alcian blue stains acid mucosubstances and acetic mucins, so it strongly stains sulfated and carboxylated mucopolysaccharides in developing cartilage. Paraformaldehyde-fixed embryos were rinsed with 70% ethanol and then stained with alcian blue solution (Sigma-Aldrich) (1% alcian blue solution in 80% ethanol/20% acetic anhydride, *w*/*w*). Embryos were rehydrated with ethanol/H_2_O series (80%, 50%, 20%, and 0% ethanol), rinsed with phosophate-buffered saline (PBS) for 15 min, and rinsed with trypsin (0.05% trypsin in sodium tetraborate, *w*/*w*, Sigma-Aldrich). Excessive dye was removed with 1% KOH/3% H_2_O_2_ (*v*/*v*) until conformation of cartilage was fully observed. For vertebrate skeletal observation, alizarin red staining was performed. Using the same fixation procedure as described above, specimens were stained with 0.1 mg/mL alizarin red in 0.5% (*w*/*w*) KOH at room temperature and destained with methanol.

### 4.6. Microinjection

Topiramate was microinjected into the embryo during the 1- to 4-cell stage. Based on the assumption of absorption of topiramate dosage in every egg laid from topiramate-treated mother fish (dose per clutch), we injected 0.5, 1.0, and 2.0 ng of topiramate between the boarder of the cell and the yolk of the embryo. Injected embryos were raised to perform analysis.

### 4.7. Statistical Analysis

The data were expressed as mean ± SD and tested by Student-*t* test. *p* < 0.05 was identified as statistically significant. 

## Figures and Tables

**Table 1:** Survival and malfunction rate of topirmate-treated offspring. Female zebrafish were continuously oral-fed with topiramate for 7 days. At the end of the final day of drug feeding, we set up a breeding environment and collected embryos the following morning and monitored the malfunction and survival that showed aberrant epiboly and early death at 5.3 and 8 hpf, respectively. Individual malfunction or survival number/total number of embryos from 19 different female zebrafish presented as individual malfunctions and survival rates. (NA: not available; hpf: hour post fertilization.)

Adult Fish	Total Number	Malfunction Number at 5.3 hpf	Survival Number at 8 hpf	Malfunction Rate at 5.3 hpf (%)	Survival Rate at 8 hpf (%)
♀31	385	7	NA	1.8	NA
♀34	419	24	NA	5.7	NA
♀52	849	147	778	17.3	91.6
♀53	412	51	NA	12.4	NA
♀54	76	17	NA	22.4	NA
♀55	64	28	NA	43.8	NA
♀56	50	30	NA	60.0	NA
♀57	178	10	NA	5.6	NA
♀58	326	96	NA	29.5	NA
♀60	14	2	NA	14.3	NA
♀61	246	40	NA	16.3	NA
♀64	287	8	282	2.8	98.3
♀65	314	91	221	29.0	70.4
♀67	212	17	189	8.0	89.2
♀73	289	NA	272	NA	94.1
♀79	317	17	308	5.4	97.2
♀80	403	30	378	7.4	93.8
♀81	326	24	279	7.4	91.1
♀82	728	38	703	5.2	96.6

**Table 2:** Survival and malfunction rate of topirmate-injected embryos. Different dosages of topiramate were injected into embryos and the malfunction and survival rates were monitored. Aberrant epiboly and early death were monitored at 5.3 and 8 hpf, respectively. Embryonic malfunction or survival number/total number of embryos from five different female zebrafish were presented as malfunction and survival rates.

Group	Injected Topiramate (ng)	Total Number	Malfunction Number at 5.3 hpf	Malfunction Rate at 5.3 hpf (%)	Survival Number at 8 hpf	Survival Rate at 8 hpf (%)
Control	0	178	0	0	170	96
	0.5	164	8	4.9	138	84
Topiramate	1	172	43	25.0	127	74
	2	101	64	63.4	60	59

## Supplementary Materials

Supplementary materials can be found at www.mdpi.com/1422-0067/18/8/1721/s1.
