# Supplementary material for: Teratogenic Effects of Topiramate in a Zebrafish Model
Source: Int J Mol Sci. 2017 Aug 7;18(8):1721. doi: 10.3390/ijms18081721 (PMC5578111; doi:10.3390/ijms18081721)
Supplement: Supplementary file 1 [file ijms-18-01721-s001.pdf]

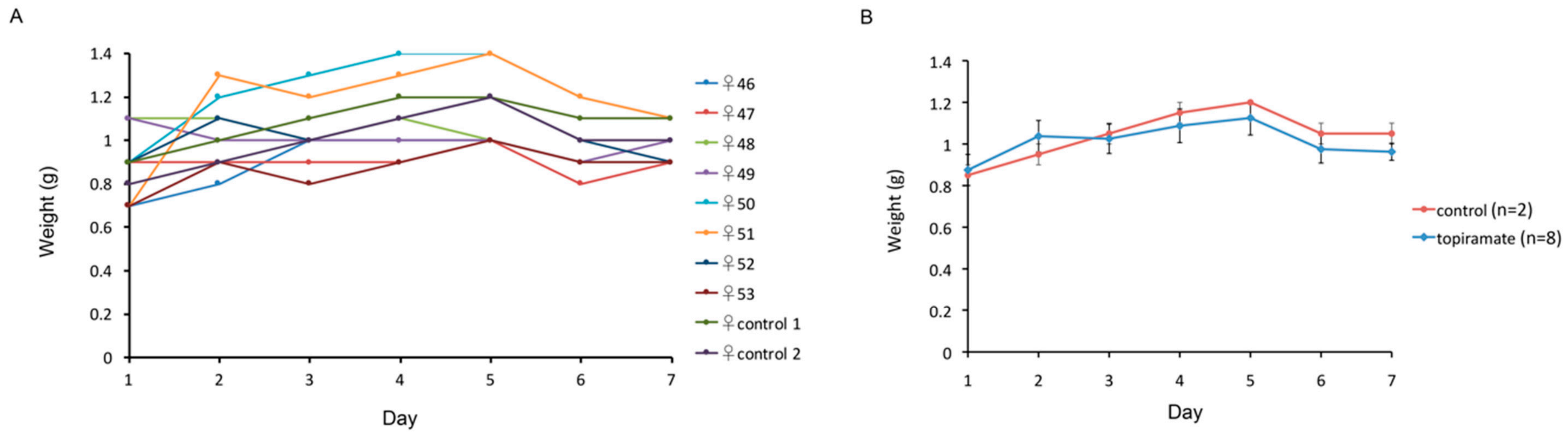

Figure S1. Body weight of control and topiramate-treated females. (A) Weight log during feeding period. (B) Statistical analysis of weight change of control and topiramate-treated group.

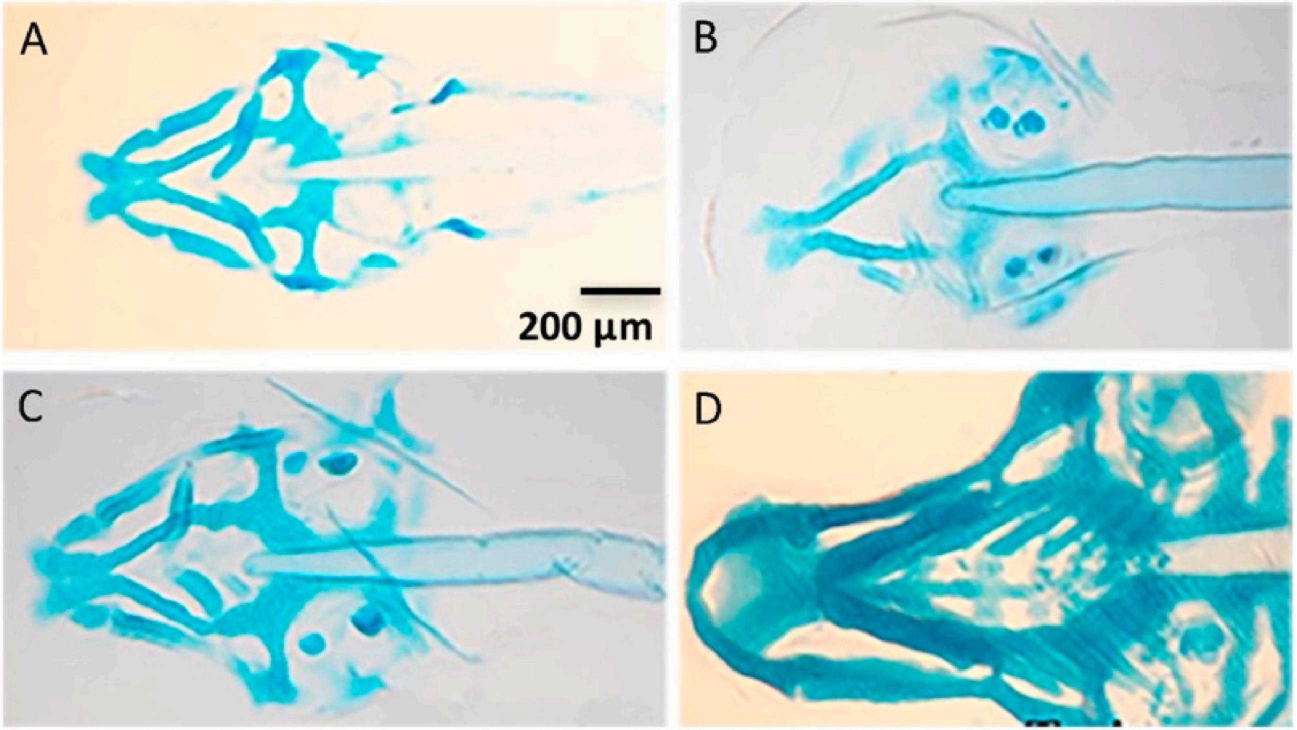

Figure S2. Craniofacial cartilage abnormalities upon diverse topiramte-treated offspring.
